# Atrial fibrillation development in the heart failure population from nationwide British linked electronic health records

**DOI:** 10.1002/ehf2.15264

**Published:** 2025-03-12

**Authors:** Hiroyuki Yoshimura, Nikhil Paliwal, Arturo Gonzalez‐Izquierdo, Chris Finan, Amand Floriaan Schmidt, Gregory Y.H. Lip, Rui Providencia

**Affiliations:** ^1^ Institute of Health Informatics Research University College London London UK; ^2^ Institute of Cardiovascular Science University College London London UK; ^3^ Centre for Health Data Science, Institute of Applied Health Research University of Birmingham Birmingham UK; ^4^ Department of Cardiology, Division Heart and Lungs University Medical Center Utrecht Utrecht The Netherlands; ^5^ Liverpool Centre for Cardiovascular Science at University of Liverpool Liverpool John Moores University and Liverpool Heart & Chest Hospital Liverpool UK; ^6^ Danish Center for Health Services Research, Department of Clinical Medicine Aalborg University Aalborg Denmark; ^7^ Barts Heart Centre, Barts Health NHS Trust London UK

**Keywords:** Atrial fibrillation, Heart failure, Electronic health record, Risk factors, Genome‐wide association study

## Abstract

**Aims:**

Atrial fibrillation (AF) is a frequent comorbidity in heart failure (HF). We analysed factors associated with new‐onset atrial fibrillation in patients with heart failure using linked real‐world UK data from primary and secondary care, along with findings from genome‐wide association studies.

**Methods and results:**

Among 163 174 participants with a diagnosis of HF (January 1998 to May 2016) from Clinical Practice Research Datalink (CPRD) and Hospital Episodes Statistics (HES), 111 595 participants had no previous history of AF (mean age 76.3 ± 12.6; 50.3% women; 95.8% white ethnicity). Multivariate weighted Cox regression was used to identify predictors for new‐onset AF. Linkage disequilibrium score regression was performed to assess the strength of the genetic correlation between AF and identified predictors. During follow‐up (median 1.33 years, IQR 0.15–4.18), the incidence rate for AF was 2.8% at 30 days, 9.9% at 1 year, 18.0% at 3 years, and 24.9% at 5 years after HF diagnosis after HF diagnosis. Female sex (HR = 0.79, 95% CI 0.71–0.88), age (HR = 1.04, 95% CI 1.04–1.04), white ethnicity (HR = 1.30, 95% CI 1.06–1.59), social deprivation (HR = 1.20, 95% CI 1.01–1.42), BMI (HR = 1.01, 95% CI 1.00–1.02), gentle physical activity (HR = 0.84, 95% CI 0.72–0.97), hypertension (HR = 1.15, 95% CI 1.03–1.29), chronic kidney disease (HR = 1.15, 95% CI 1.06–1.24), chronic obstructive pulmonary disease (COPD) (HR = 1.10, 95% CI 1.01–1.19) and valvular heart disease (HR = 1.17, 95% CI 1.06–1.29) were associated with new‐onset AF. Angiotensin‐converting enzyme inhibitors were associated with lower AF incidence (HR = 0.88, 95% CI 0.80–0.96), and the magnitude of effect was dependent on the duration of administration. Linkage disequilibrium score regression showed important genetic correlation between AF and HF (rg = 0.57, *P* = 2.30 × 10^‐59^) and reduced, but still significant, overlap between AF and BMI (rg = 0.19, P = 6.18 × 10^‐20^), systolic and diastolic blood pressure, smoking, and COPD (*P* values ranging from <10^‐4^ to <0.05).

**Conclusions:**

Incident AF in the HF population is high, with good genetic correlation for the two conditions. Identified predictors for new‐onset AF might be helpful to improve management of HF patients and AF prevention.

## Introduction

Atrial fibrillation (AF) and heart failure (HF) are two major cardiovascular diseases whose prevalence has increased over recent decades and is expected to increase further due to the ageing of the population.^1^, ^2^ It is well known that HF can increase the risk of developing AF, while AF can lead to HF.^3^ Both conditions confer clinical complexity, with share (often multiple) risk factors, such as hypertension, diabetes, ageing, and obesity.^4^, ^5^ Also, patients with both HF and AF have worse prognosis than patients with AF or HF alone.^6^, ^7^

The European Society of Cardiology (ESC) guidelines highlight a role for upstream therapy, drugs not classified as anti‐arrhythmic agents, for preventing the occurrence or recurrence of AF through modification of the atrial substrate or targeting AF‐specific mechanisms.^8^ A meta‐analysis of 23 randomized controlled trials suggested that patients treated with angiotensin‐converting enzyme inhibitor (ACEi)/angiotensin receptor blockers (ARBs) had significantly lower incidence of AF versus those not treated with these drugs.^9^

Although the ESC and American Heart Association (AHA)/American College of Cardiology (ACA)/Heart Rhythm Society (HRS) guidelines summarized the factors associated with new‐onset AF in the general population, these factors may not apply to the HF population.^8^, ^10^, ^11^ Predictors of new‐onset AF in the HF population have been investigated in a Korean Acute Heart Failure registry, but due to regional and ethical differences in AF rates and associated factors, it is uncertain if such findings would be applicable in a European population.^2^, ^6^, ^8^ A Swedish Heart Failure Registry assessed the association of HF with prevalent AF but did not analyse new‐onset AF following HF or important drivers of the association such as ethnicity, chronic obstructive pulmonary disease (COPD), or social deprivation.^12^

Additional insights come from genetic associations. Genome‐wide association study (GWAS) have identified over 150 AF‐related loci, providing basis for gaining understanding of the underlying AF mechanisms and associations with risk factors, and comorbidities like HF.^13^

In this study, we aimed to assess potential risk and protective factors for new‐onset AF in the UK's HF population. Understanding the predictors of new‐onset AF for HF patients could lead to better management of HF patients, prevent arrhythmia development, and improve their outcomes. To the best of our knowledge, no previous studies have investigated the factors for new‐onset AF for the HF population using linked UK primary and secondary care datasets and publicly available data from GWAS. Doing this, using UK real‐world clinical data, will help to better address the needs and optimize prevention in the UK population and populations with similar ethnic and social representation.

## Methods

### Data sources

We linked three of UK's nationwide electronic health records (EHR) databases: primary care records from the Clinical Practice Research Datalink (CPRD), secondary care records from the Hospital Episodes Statistics (HES), and death census from the Office of National Statistic (ONS).^14^

To identify comorbidities, READ and/or SNOMED CT codes in the CPRD dataset, and International Classification of Diseases, Tenth Revision (ICD‐10) code in the HES and ONS datasets were used. Therapy information was recorded in British National Formulary (BNF) code. *Table* *S1* illustrates the utilized clinical codes lists to identify comorbidities. These have been previously described, and their validity has been confirmed using a systematic validation framework for evaluating accuracy.^15^

### Study population

**Figure 1:**
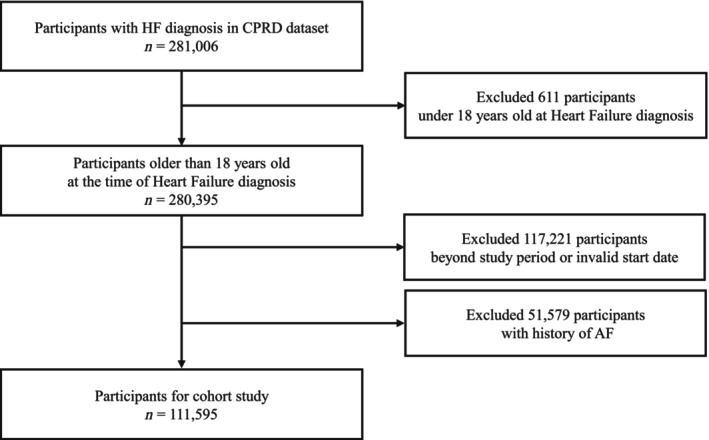
Study flow chart.

The study population was enrolled from 1 January 1998 to 31 May 2016. The participants in the CPRD dataset who were not linked to HES and were under 18 years old were excluded. After excluding them, CPRD contained 6 529 382 participants. *Figure* *1* illustrates the flowchart of the study population. Among 281 006 diagnosed HF participants in primary and secondary care, 163 174 participants were enrolled after removing participants diagnosed beyond the study periods and under 18 years old at HF diagnosis. After removing 51 579 participants whose AF diagnosis date was before the HF diagnosis date, 111 595 participants were enrolled in the cohort study. AF was diagnosed in 15 879 participants on the same date of HF diagnosis. Patients were followed up from HF diagnosis date to the earliest date of the following: occurrence of AF diagnosis, death, transfer out, or end of the study period.

### Variables

This analysis included factors associated with new‐onset AF reported by ESC guideline,^8^ alongside additional risk factors identified following non‐structured literature reviews, such as age, gender, ethnicity, social deprivation, body mass index (BMI), smoking, alcohol, physical activity, hypertension, chronic kidney disease (CKD), sleep apnoea, COPD, ischaemic heart disease (IHD), valvular heart disease, dyslipidaemia, and stroke.^8^ The comorbidities and treatments included among these variables are commonly reported in routine cardiovascular care.

Ethnicity was classified into two categories: White and non‐White. Alcohol variable included never drinker and drinker, defined as those without and with any drinking records before the HF diagnosis, respectively. For the smoking variable, never smoker referred to those without any smoking records prior to HF diagnosis, while former and current smoker were identified from the closest records from the HF diagnosis date. The physical activity variable in the CPRD dataset was categorized into four levels: inactive, gentle, moderate, and vigorous. We assessed the index of multiple deprivation, a measure of quantify relative deprivation (essentially a measure of poverty) of small areas across the country, classifying them into five quintiles (from most to least deprived). For the purpose of our analyses, we assessed the impact of living in the most deprived socio‐economic quintile areas.

Baseline medication was assessed at the time of HF diagnosis. For the analyses on ACEi, ARB, and MRA treatment duration and risk of new‐onset AF, medical therapy during follow‐up was assessed. The duration of treatments was calculated as the total number of prescription days after HF diagnosis. Any treatment use was defined as having at least one prescription record during the follow‐up period, while no treatment was defined as the absence of any prescription records.

### Statistical analysis

For the baseline characteristics, we reported the number and proportion (%) for categorical variables and means and standard deviation for continuous variables. This study consists of a retrospective cohort study including an HF population with no previous history of AF.

The analyses were multivariate Cox regression models with complete case analysis to evaluate the association between new‐onset AF and risk and protective factors. The proportional hazards assumption was assessed by Schoenfeld residuals against the transformed time. When the proportional hazards assumption was violated, weighted Cox regression model was used to estimates unbiased average hazard ratio (HR) irrespective of hazard proportionality.^16^ This weighting function at each event time reflects the expected number of subjects at risk adjusted for censoring. In our analysis, several variables in multivariate Cox regression were violated in hazard assumption.

The multivariate regressions were adjusted demographic and comorbidity‐related variables (gender, age, ethnicity, deprivation, BMI, smoking, alcohol, physical activity, hypertension, CKD, diabetes, sleep apnoea, COPD, IHD, valvular heart disease, dyslipidaemia, and stroke). As a sensitivity analysis, we assume the missing values are missing at random and imputed missing values by random forest imputation. All statistical analyses were performed using R 3.6.2 and RStudio 1.2.

### Assessment of genetic overlap from genome‐wide association study

With the aim of providing further validation to the predictors identified in the previous analysis, we assessed the genetic correlation between AF, heart failure, and the following traits: hypertension/blood pressure levels, exercise, BMI, smoking, and COPD. We analysed genome‐wide single‐nucleotide polymorphism (SNP) data and extracted full summary statistics from GWAS identified from GWAS catalogue^17^ and performed linkage disequilibrium (LD) score regression to quantify the strength of the genetic correlation (r_g_) between AF and other traits. LD score regression operates on the principle that SNPs with higher LD tend to capture more heritable variance. By regressing the GWAS effect sizes of each SNP on its LD score, the method distinguishes true genetic overlap from confounding factors such as population stratification, relatedness, and other biases inherent to GWAS data. This approach allows for robust estimates of genetic correlation, enabling insights into shared molecular pathways and aetiological mechanisms across traits. We followed the previously described approach to implement LD score regression analysis.^18^, ^19^, ^20^

A list of studies for whom summary statistics was downloaded is presented in *Table* *S2*.

### Ethical approval

The protocol was approved by the Independent Scientific Advisory Committee of the Medicines and Healthcare products Regulatory Agency (the protocol 17_205R).

## Results

*Table* *1* demonstrates the baseline characteristics of the population. Among 111 595 participants without AF at baseline enrolled (mean age 76.3 ± 12.6; 50.3% women; 95.8% white ethnicity), 19 628 participants (17.6%) developed new‐onset AF over a median follow‐up of 1.33 years (IQR 0.15–4.18). The demographic and comorbidity profiles of participants with no AF and new‐onset AF were similar. All treatments except mineralocorticoid receptor antagonist (MRA) were more frequently used at baseline for participants who progressed to develop new‐onset AF (vs. those that did not develop AF).

**Table 1:** Baseline characteristics.

Characteristic	Overall, *n* (%)	No AF, *n* (%)	New‐onset AF, *n* (%)
*N*	111 595	91 967	19 628
Women	56 091 (50.3%)	46 681 (50.8%)	9410 (47.9%)
Age^a^	76.3 (12.6)	76.2 (12.9)	76.4 (10.7)
White ethnicity^b^	95 580 (95.8%)	77 472 (95.6%)	18 108 (96.9%)
Most deprived quintile	15 455 (13.8%)	12 711 (13.8%)	2744 (14.0%)
BMI^a^ ^,^ ^b^	27.5 (6.2)	27.4 (6.2)	27.9 (6.1)
Smoking^b^			
Never smoker	35 734 (35.3%)	29 186 (35.2%)	6548 (35.5%)
Former smoker	38 308 (37.8%)	31 117 (37.5%)	7191 (39.0%)
Current smoker	27 328 (27.0%)	22 613 (27.3%)	4715 (25.6%)
Alcohol^b^	36 586 (61.6%)	29 768 (61.3%)	6818 (62.8%)
Physical activity^b^			
Inactive	11 191 (20.4%)	9039 (20.6%)	2152 (19.4%)
Gentle	27 100 (49.4%)	21 413 (48.9%)	5687 (51.3%)
Moderate	15 677 (28.6%)	12 602 (28.8%)	3075 (27.7%)
Vigorous	910 (1.7%)	732 (1.7%)	178 (1.6%)
Hypertension	68 515 (61.4%)	55 925 (60.8%)	12 590 (64.1%)
CKD	14 539 (13.0%)	12 091 (13.1%)	2448 (12.5%)
Diabetes	23 667 (21.2%)	19 725 (21.4%)	3942 (20.1%)
Sleep apnoea	1427 (1.3%)	1212 (1.3%)	215 (1.1%)
COPD	54 631 (49.0%)	44 971 (48.9%)	9660 (49.2%)
Ischaemic heart disease	45 073 (40.4%)	36 865 (40.1%)	8208 (41.8%)
Valvular heart disease	14 856 (13.3%)	11 928 (13.0%)	2928 (14.9%)
Dyslipidaemia	26 629 (23.9%)	21 808 (23.7%)	4821 (24.6%)
Stroke	13 154 (11.8%)	11 300 (12.3%)	1854 (9.4%)
CHA2DS2‐VASc			
1	2891 (2.6%)	2464 (2.7%)	427 (2.2%)
2	8714 (7.8%)	7379 (8.0%)	1335 (6.8%)
3	16 990 (15.2%)	13 926 (15.1%)	3064 (15.6%)
4	27 544 (24.7%)	22 442 (24.4%)	5102 (26.0%)
5	28 808 (25.8%)	23 506 (25.6%)	5302 (27.0%)
6	16 642 (14.9%)	13 729 (14.9%)	2913 (14.8%)
7	6875 (6.2%)	5850 (6.4%)	1025 (5.2%)
8	2564 (2.3%)	2,178 (2.4%)	386 (2.0%)
9	567 (0.5%)	493 (0.5%)	74 (0.4%)
ACEi	52 361 (46.9%)	42 177 (45.9%)	10 184 (51.9%)
ARB	13 958 (12.5%)	11 271 (12.3%)	2687 (13.7%)
Beta‐blocker	46 265 (41.5%)	37 154 (40.4%)	9111 (46.4%)
MRA	6734 (6.0%)	5646 (6.1%)	1088 (5.5%)
Antiplatelet	59 044 (52.9%)	48 172 (52.4%)	10 872 (55.4%)
Diuretics	78 343 (70.2%)	63 673 (69.2%)	14 670 (74.7%)
NSAIDs	69 013 (61.8%)	56 382 (61.3%)	12 631 (64.4%)
Statin	41 002 (36.7%)	33 368 (36.3%)	7634 (38.9%)

*Note*: *P* values for all comparisons of no AF versus new‐onset AF or prevalent AF are <0.001.

Abbreviations: ACEi, angiotensin‐converting enzyme inhibitors; ARB, angiotensin receptor blockers; BMI, body mass index; CKD, chronic kidney disease; COPD, chronic obstructive pulmonary disease; MRA, mineralocorticoid receptor antagonists; NSAID, non‐steroidal anti‐inflammatory drugs.

^a^ Mean (SD).

^b^ Missing values were observed in several variables, with 10.6% in ethnicity, 17.4% in BMI, 9.2% in smoking, 46.8% in alcohol, and 50.8% in physical activity.

During follow‐up, 69 919(65.8%), 21 242(20.0%), and 20 587(19.4%) participants received at least one dose of ACEi, ARB, and MRA, respectively.

### Atrial fibrillation incidence for newly diagnosed heart failure population

**Figure 2:**
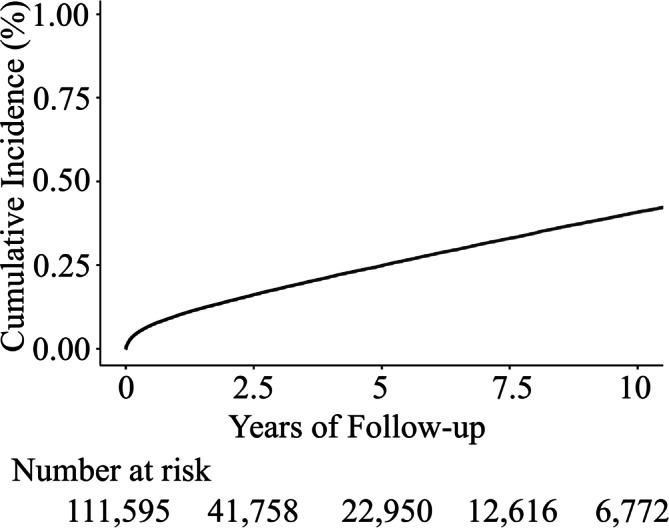
Kaplan–Meier plot for new‐onset atrial fibrillation after a new heart failure diagnosis.

The incidence rate of AF was 2.8% at 30 days, 9.9% at 1 year, 18.0% at 3 years, and 24.9 at 5 years after HF diagnosis (*Figure* *2*).

The weighted multivariate Cox regression models demonstrated a lower risk of new‐onset AF for females (HR = 0.79, 95% confident interval [CI] 0.71–0.88) and patients involved in gentle physical activity (HR = 0.84, 95% CI 0.72–0.97) (*Table* *2*). Participants who were older, White ethnicity, in the most deprived strata, and with higher BMI were more likely to develop new‐onset AF (HR = 1.04, 95% CI 1.04–1.04; HR = 1.30, 95% CI 1.06–1.59; HR = 1.20, 95% CI 1.01–1.42; HR = 1.01, 95% CI 1.00–1.02, respectively). Participants with hypertension, CKD, COPD, and valvular heart disease experienced an increase in new‐onset AF risks of 15% (95% CI 1.03–1.29), 15% (95% CI 1.06–1.24), 10% (95% CI 1.01–1.19), and 17% (95% CI 1.06–1.29), respectively.

**Table 2:** Analysis for predictors of new‐onset AF.

Characteristics	Unadjusted HR (95% CI)	Adjusted HR (95% CI)
Women	0.95 (0.86, 1.05)	0.79 (0.71, 0.88)
Age	1.04 (1.03, 1.04)	1.04 (1.04, 1.04)
White ethnicity	1.50 (1.23, 1.82)	1.30 (1.06, 1.59)
Most deprived quintile	1.25 (1.05, 1.50)	1.20 (1.01, 1.42)
BMI	1.00 (0.99, 1.00)	1.01 (1.00, 1.02)
Smoking		
Never smoker	Ref	Ref
Former smoker	0.94 (0.76, 1.17)	0.87 (0.70, 1.07)
Current smoker	0.77 (0.62, 0.96)	0.83 (0.67, 1.03)
Alcohol	1.05 (0.95, 1.15)	1.03 (0.94, 1.13)
Physical activity		
Inactive	Ref	Ref
Gentle	0.86 (0.74, 0.99)	0.84 (0.72, 0.97)
Moderate	0.81 (0.69, 0.95)	0.85 (0.72, 1.02)
Vigorous	0.75 (0.52, 1.10)	0.86 (0.59, 1.26)
Hypertension	1.31 (1.18, 1.46)	1.15 (1.03, 1.29)
CKD	1.47 (1.38, 1.58)	1.15 (1.06, 1.24)
Diabetes	1.02 (0.95, 1.10)	1.03 (0.94, 1.12)
Sleep apnoea	1.00 (0.75, 1.33)	1.17 (0.88, 1.56)
COPD	1.09 (1.00, 1.20)	1.10 (1.01, 1.19)
Ischaemic heart disease	0.99 (0.90, 1.09)	0.99 (0.89, 1.10)
Valvular heart disease	1.17 (1.06, 1.30)	1.17 (1.06, 1.29)
Dyslipidaemia	1.02 (0.93, 1.12)	1.06 (0.95, 1.18)
Stroke	1.07 (0.97, 1.20)	0.92 (0.83, 1.03)

*Note*: 95% CI, 95% confident interval; BMI, body mass index; CKD, chronic kidney disease; COPD, chronic obstructive pulmonary disease; HR, hazard ratio.

**Figure 3:**
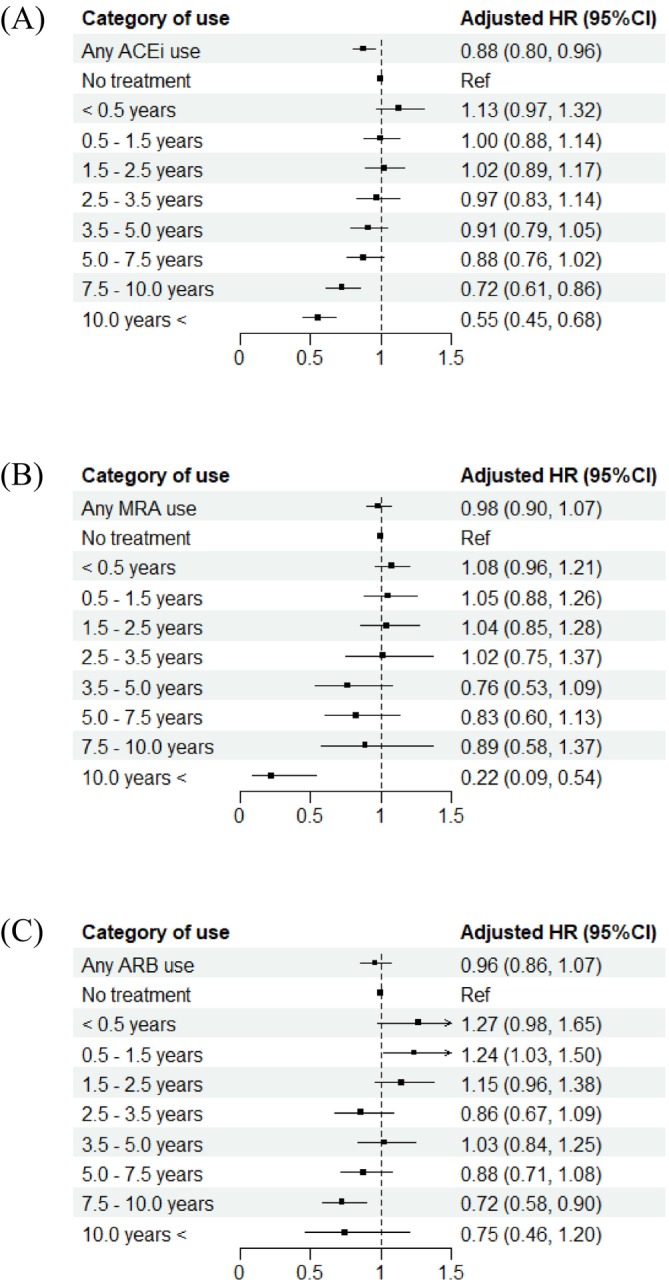
Analysis of treatment duration on the risk of new‐onset AF: (A) ACEi; (B) ARB; (C) MRA. Note: During follow‐up, 24 069 (75.9%), 8380 (26.4%), and 6965 (22.0%) participants received at least one dose of ACEi, ARB, and MRA, respectively. ACEi, angiotensin‐converting enzyme inhibitors; ARB, angiotensin receptor blockers; MRA, mineralocorticoid receptor antagonists.

Treatment with ACEi after HF diagnosis was associated with lower risk of new‐onset AF (HR = 0.88, 95% CI 0.80–0.96). As compared with no ACEi treatment, longer treatment duration of ACEi was associated with lower new‐onset AF risk (*Figure* *3*). A trend for reduction of new‐onset AF with ARB and MRA was not observed (HR = 0.98, 95% CI 0.90–1.07, HR = 0.96, 95% CI 0.86–1.07, respectively).

### Sensitivity analysis

The analysis for the imputed dataset showed same trend of association of new‐onset AF except deprivation (HR = 1.04, 95% CI 0.96–1.13), moderate physical activity (HR = 0.91, 95% CI 0.83–0.99), diabetes (HR = 1.06, 95% CI 1.00–1.12), and stroke (HR = 0.93, 95% CI 0.87–0.99) (*Table* *S3*). While the same trends for association with new‐onset AF and duration of ACEi and MRA treatment, any ARB use was associated with lower new‐onset AF by 6% (95% CI 0.88–1.00), and the benefit became more apparent with longer treatment duration (*Figure* *S1*).

### Genetic correlation between atrial fibrillation, heart failure, and predictors

**Figure 4:**
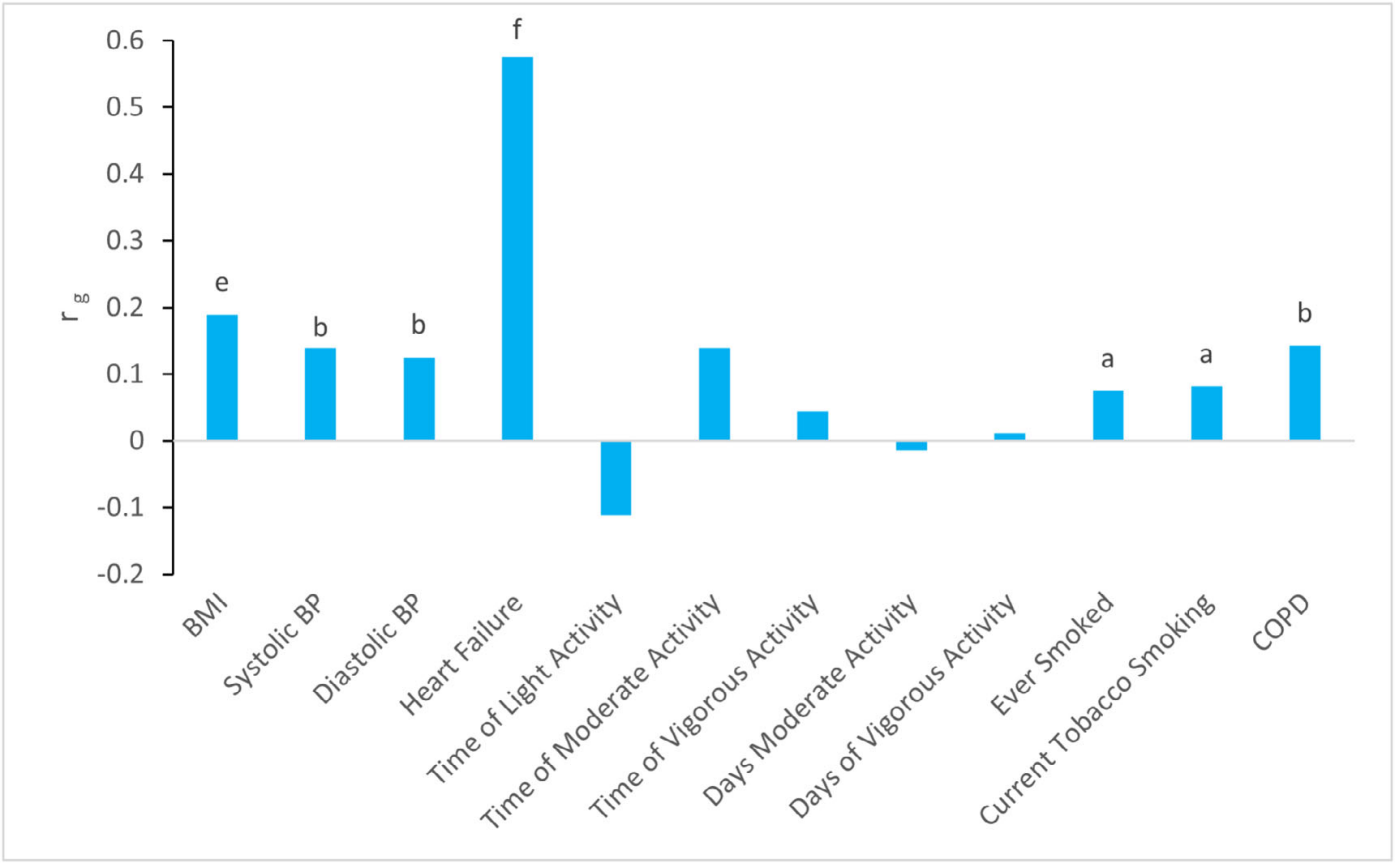
Genetic correlation between AF, heart failure and risk factors. Legend: significance of results: a = *P* < 0.05; b = *P* < 10^−4^; c = *P* < 10^−8^; d = *P* < 10^−12^; e = *P* < 10^−16^; f = *P* < 10^−50^. More information on coefficient levels is available in *Table* *S4*. BMI, body mass index; BP, blood pressure; COPD, chronic obstructive pulmonary disease.

*Figure* *4* illustrates the genetic correlation between AF and the different traits we assessed. We observed highly significant genetic correlations, with the strongest overlap between AF and heart failure (r_g_ = 0.57, *P* = 2.30 × 10^−59^), suggesting a substantial shared genetic basis. Similarly, BMI showed a significant correlation (r_g_ = 0.19, *P* = 6.18 × 10^−20^), reinforcing the influence of metabolic traits on AF susceptibility. These findings indicate that traits with moderate or significant genetic overlap, such as systolic and diastolic blood pressure, COPD, and smoking (*P*‐ alues ranging from <10^−4^ to <0.05), may share underlying molecular mechanisms with AF.

## Discussion

We described the association of AF with HF in the UK population and, to the best of our knowledge, present the first European report on predictors for new‐onset AF following an HF diagnosis from EHR and GWAS. Our principal findings are as follows: (i) The subsequent rate of new‐onset AF was 2.8% at 30 days and 9.9% at 1 year after HF diagnosis, with a quarter of patients developing AF in the first 5 years after the HF diagnosis, providing support to the close association between the two entities (almost 40% of HF patients have or will develop AF at some point); (ii) we identified an important association between ethnicity, deprivation, and AF incidence, which had not been previously reported in previous European EHR studies; and (iii) we observed an important genetic correlation between AF and HF. Weaker correlation, but meeting *P*‐value cut‐offs for significance, was observed for some of the identified predictors in the epidemiology study: BMI, systolic and diastolic blood pressure, smoking, and COPD.

The risk factors for new‐onset AF identified in our study overlap to some extent with those in guidelines for the general population and previous publications.^6^, ^8^, ^12^ Our study, which provides a more detailed description of the temporal association between AF and HF and duration of exposure to treatment, showed that use of ACEi, and possibly ARB, was associated with lower risk of new‐onset AF with benefit more pronounced for patients with longer treatment.^6^, ^12^ These findings have been replicated in other cohorts, namely, in Taiwan.^21^ The higher risk of new‐onset AF within the initial 6 months of treatment with ACEi after a diagnosis of HF may reflect more advanced forms of HF, more likely to be started on treatment, and also more likely to develop AF within a short timeframe. Importantly, we obtained similar findings in our ARB analyses. Certainty of our primary analysis was lower due to the smaller sample size, but after imputing missing data (i.e. and increasing sample size), a comparable trend with higher risk of new‐onset AF in the initial 6 months of treatment was also observed for ARB. The duration of HF treatment may be associated with the severity of HF. Previous studies have reported low prescription rates and suboptimal dosing of HF treatments for patients with severe HF due to adverse events.^22^ Additionally, high discontinuation rates of HF therapy have also been observed.^23^

Previous registries have reported a low uptake of guideline‐directed medical therapy (GDMT) for HF.^22^, ^24^ The CHAMP‐HF Registry showed that 27% and 67% of patients were not prescribed ACEi/ARB/ARNI and MRA, respectively. Additionally, only 17%, 14%, and 77% of patients received target doses of ACEi/ARB, ARNI, and MRA, respectively.^24^ The HELP‐HF registry demonstrated low rates of GDMT, and GDMT was associated with lower mortality and HF hospitalization.^22^ Our real‐world UK data spanning from 1998 to 2016 also showed a suboptimal uptake of HF treatments (ACEi: 65.8%, ARB: 20.0%, MRA: 19.4%), which might have affected the incidence of AF. Use of ACEi, ARB, and MRA in heart failure patients treated in the United Kingdom has increased between 2001 and 2012, suggesting that the low uptake of these drugs in our cohort may be driven by underutilization in the earlier years.^25^

Our findings highlight varying degrees of genetic overlap between AF and traits such as blood pressure/hypertension, smoking, COPD, and BMI, with the most pronounced overlap observed for heart failure (r_g_ = 0.57). This suggests that AF shares a significant proportion of its genetic basis with HF, potentially reflecting common molecular mechanisms, such as pathways involving myocardial remodelling or neurohormonal regulation.^13^, ^26^ Similarly, BMI (r_g_ = 0.19) and blood pressure traits show moderate overlap, aligning with previous studies that have linked metabolic and hemodynamic factors to AF susceptibility.^26^ The use of LD score regression in this study provided several advantages. By accounting for LD patterns and confounders like population stratification, the method allowed for precise quantification of shared heritability. This ensures that the observed genetic correlations are not merely artefacts of GWAS biases but reflect meaningful biological connections. The observed genetic correlations also provide a basis for identifying pleiotropic genes and pathways that contribute to multiple traits. For example, the strong overlap between AF and HF reinforces the possibility for integrated therapeutic strategies targeting shared pathways. Our analyses of associated genetic variants provide validation to our cohort study findings, supporting the intensive management of the identified risk factors and providing a rationale for assessment of other drug repurposing options. A previous report shows that genetically predicted blood pressure levels and hypertension associate with higher risk of AF.^27^ In addition, the prespecified secondary analysis of CHARM programme, which is a randomized control trial (RCT), reported ARB treatment was associated with lower AF incidence for chronic HF population.^28^ This aligns with our data suggesting a reduction of new onset of AF in the HF population for patients treated with ACEi.

### Clinical implications

As far as we are aware, this is the first study on identifying the risk factors for new‐onset AF incidence in HF patients using UK nationwide EHR and publicly available data from GWAS. These results may help to understand the factors associated with new‐onset AF for HF patients in the United Kingdom and other countries with a similar demographic and ethnic structure. Since the concomitance of AF and HF increases mortality, preventing AF in HF patients is essential for improving the outcomes for HF patients. Treatment and reduction of AF burden in the HF population have been shown to reduce mortality.^29^

These results also raise awareness for new‐onset AF risks factors and encourage healthcare providers to diagnose them and aggressively intervene in their early stages. Previous studies suggested the possibility that modifying risk factors, such as obesity, smoking, and hypertension, could reduce the AF risk.^30^ In addition, our findings support the need of AF patients to adhere to the Atrial Fibrillation Better Care (ABC) pathway with implementation of lifestyle changes to improve AF risk status.^8^, ^31^, ^32^

### Limitations

A few limitations merit further discussion. First, as for every EHR study, there is a risk for unmeasured confounders, some of which might be associated with new‐onset AF. For example, a previous registry suggested that prevalence of AF was different among the types of HF, such as heart failure with preserved ejection fraction (HFpEF), mid‐range ejection fraction (HFmrEF), and reduced ejection fraction (HFrEF), and higher ejection fraction significantly increased the prevalence of AF.^33^ Second, the granularity of data is also one of the limitations, meaning that detailed information for some variables (e.g. height, smoking, alcohol, and dietary factors) was not routinely collected. Granularity on medical therapy (i.e. dose changes) is also not available. Essential data to classify the type of HF and to determine the optimal treatment such as symptoms, signs, echocardiographic data and blood test data are missing. However, this study included potential risk and protective factors described in the ESC guidelines, as well as essential comorbidities and treatments recorded in routine cardiovascular care. Third, new HF treatments have been routinely implemented in routine HF practice after the study period, such as the angiotensin receptor/neprilysin inhibitor (ARNi) and the sodium‐glucose cotransporter‐2 (SGLT2) inhibitors; this dataset included most guideline‐recommended treatments, namely, those that were used at the time of the study.^8^ Fourth, our dataset has no information on doses of HF medication. We acknowledge that these are real‐world data including patients on maximal tolerated dose, and possibly also patients also on suboptimal doses. Fifth, there is a possibility of generating collider bias by analysing only HF patients. However, predictors and risk factors for new‐onset AF in the general population have been previously investigated.^34^ Segan *et al*. identified hypertension, age, BMI, male sex, sleep apnoea, smoking, and alcohol.^34^ Except for the last three risk factors, the remaining were reproduced by our findings, and we identified five additional factors not present in this UK Biobank and Framingham study. Interestingly, these two cohorts have a very detailed characterization of diet, smoking and alcohol intake, which raises the question of whether this was not captured in our simpler EHR categorization or if due to reverse causation. On the other hand, an analysis of the CPRD dataset for new‐onset predictors of AF in the general UK population identified was more aligned with our findings.^35^ Finally, some variables had missing values (e.g. smoking, BMI, etc.). However, the sensitivity analyses in imputed datasets showed similar direction of association for new‐onset AF, indicating the robustness of our findings. Furthermore, data missingness and validity of multiple imputation for the CPRD dataset have been previously assessed, namely, for BMI and smoking, and their results suggest that multiple imputation provides unbiased results.^36^

## Conclusions

We showed a strong association of AF and HF in the UK population and a quarter of patients without previously known AF, having a diagnosis of new‐onset AF over the next 5 years. Factors such as male sex, older age, White ethnicity, deprivation, higher BMI, physical inactivity, hypertension, CKD, COPD, and valvular heart disease increased the risk of new‐onset AF. ACEi treatment was associated with the lower risk of new‐onset AF, with more pronounced benefit in patients treated for longer time. Further studies are required to validate our findings, with assessment of causality, and to investigate the impact of addressing the identified risk factors for the prevention of AF in the HF population.

## Conflict of interest

GYHL is a consultant and speaker for BMS/Pfizer, Boehringer Ingelheim, Daiichi‐Sankyo, and Anthos. No fees are received personally. No other conflicts of interest to declare.

## Funding

RP is supported by the University College London British Heart Foundation Research Accelerator (grant number AA/18/6/34223) and National Institute of Health Research (grant number NIHR129463). AFS is supported by British Heart Foundation grant numbers PG/18/5033837 and PG/22/10989 and the University College London British Heart Foundation Research Accelerator AA/18/6/34223. GYHL is co‐principal investigator of the AFFIRMO project on multimorbidity in AF, which has received funding from the European Union's Horizon 2020 research and innovation programme under grant agreement number 899871.

## Supporting information


**Table S1.** Codes for comorbidities.
**Table S2.** GWAS summary statistics for each trait.
**Table S3.** Sensitivity analysis.
**Table S4.** Results of linkage disequilibrium score regression.
**Figure S1.** Sensitivity analysis of treatment duration on the risk of new‐onset AF: A. ACEi; B. ARB; C. MRA.
