# Supplementary material for: Atrial fibrillation development in the heart failure population from nationwide British linked electronic health records
Source: ESC Heart Fail. 2025 Mar 12;12(4):2507–17. doi: 10.1002/ehf2.15264 (PMC12287870; doi:10.1002/ehf2.15264)
Supplement: Supplementary file 1 — Table S1. Codes for comorbidities. Table S2. GWAS summary statistics for each trait. Table S3. Sensitivity analysis. Table S4. Results of linkage disequilibrium score regression. Figure S1. Sensitivity analysis of treatment duration on the risk of new‐onset AF: A. ACEi; B. ARB; C. MRA. [file EHF2-12-2507-s001.docx]

**Atrial Fibrillation development in the Heart Failure population**

**from Nationwide British linked-electronic health records**

**Supplemental materials**

[**Supplemental Table 1.** Codes for comorbidities 2](#_Toc177644342)

[**Supplemental Table 2.** GWAS summary statistics for each trait 3](#_Toc177644343)

[**Supplemental Table 3.** Sensitivity analysis 4](#_Toc177644344)

[**Supplemental Table 4.** Results of linkage disequilibrium score regression 5](#_Toc177644345)

[**Supplemental Figure 1.** Sensitivity analysis of treatment duration on the risk of new-onset AF: A. ACEi; B. ARB; C. MRA. 7](#_Toc174004927)

**Supplemental Table 1.** Codes for comorbidities

| **Variable** | **Codes list** |
| --- | --- |
| **Heart failure** | http://old.caliberresearch.org/portal/show/hf_gprd (Category 3, 4, 5, 6) |
|  | http://old.caliberresearch.org/portal/show/hf_hes (Category 4, 6) |
| **Atrial fibrillation** | http://old.caliberresearch.org/portal/show/af_gprd (Category 3, 4, 5, 6, 7) |
|  | http://old.caliberresearch.org/portal/show/af_hes |
| **Smoking status** | http://old.caliberresearch.org/portal/show/smoking_status_gprd |
|  | <http://old.caliberresearch.org/portal/show/smoking_status_hes> |
| **Alcohol** | http://old.caliberresearch.org/portal/show/alcohol_drinker_gprd |
| **Physical activity** | <http://old.caliberresearch.org/portal/show/physact_gprd> |
| **Hypertension** | http://old.caliberresearch.org/portal/show/ht_gprd (Category 3, 4) |
|  | http://old.caliberresearch.org/portal/show/ht_hes (Category 3, 4) |
| **Chronic kidney disease** | <http://old.caliberresearch.org/portal/show/ckdstage_gprd> |
| **Diabetes** | http://old.caliberresearch.org/portal/show/dm_gprd (Category 3, 4, 6) |
|  | http://old.caliberresearch.org/portal/show/dm_hes (Category 3, 4, 6) |
| **Sleep apnoea** | http://old.caliberresearch.org/portal/show/obstruct_sleep_apnoea_gprd |
|  | http://old.caliberresearch.org/portal/show/obstruct_sleep_apnoea_hes |
| **Chronic obstructive pulmonary disease** | http://old.caliberresearch.org/portal/show/copd_gprd |
|  | http://old.caliberresearch.org/portal/show/copd_hes |
| **Ischemic heart disease** | http://old.caliberresearch.org/portal/show/chest_pain_gprd (Category 4) |
|  | http://old.caliberresearch.org/portal/show/sa_diagnosis_gprd (Category 4) |
|  | http://old.caliberresearch.org/portal/show/angina_hes (Category 4) |
|  | http://old.caliberresearch.org/portal/show/unangina_gprd (Category 3) |
|  | http://old.caliberresearch.org/portal/show/acs_gprd (Category 3) |
|  | http://old.caliberresearch.org/portal/show/uangina_hes |
|  | http://old.caliberresearch.org/portal/show/acute_ihd_hes |
|  | http://old.caliberresearch.org/portal/show/myo_infarct_gprd (Category 3, 4, 5) |
|  | http://old.caliberresearch.org/portal/show/myo_infarct_hes (Category 5) |
| **Valvular heart disease** | http://old.caliberresearch.org/portal/show/cardiacvalve_gprd |
|  | http://old.caliberresearch.org/portal/show/cardiacvalve_hes |
| **Dyslipidaemia** | http://old.caliberresearch.org/portal/show/dyslipid_gprd |
|  | http://old.caliberresearch.org/portal/show/dyslipid_hes |
| **Stroke** | http://old.caliberresearch.org/portal/show/ischaemic_stroke_gprd (Category 3) |
|  | http://old.caliberresearch.org/portal/show/ischaemic_stroke_hes (Category 3) |
|  | http://old.caliberresearch.org/portal/show/haem_stroke_gprd (Category 3) |
|  | http://old.caliberresearch.org/portal/show/haem_stroke_hes (Category 3) |
|  | http://old.caliberresearch.org/portal/show/stroke_nos_gprd (Category 3) |
|  | http://old.caliberresearch.org/portal/show/stroke_nos_hes (Category 3) |
|  | http://old.caliberresearch.org/portal/show/stroke_nos_opcs (Category 3) |

**Supplemental Table 2.** GWAS summary statistics for each trait

| **Variable** | **URL** |
| --- | --- |
| **Heart failure^1^** | https://pubmed.ncbi.nlm.nih.gov/31919418/ |
| **Atrial fibrillation^2^** | https://pubmed.ncbi.nlm.nih.gov/30061737/ |
| **Body mass index^3^** | https://pubmed.ncbi.nlm.nih.gov/30239722/ |
| **Systolic / Dilated blood pressure^4^** | https://pubmed.ncbi.nlm.nih.gov/38689001/ |
| **COPD/ Activity status/ Smoking status** | http://www.nealelab.is/uk-biobank/ |

**References**

1. Shah S, Henry A, Roselli C, Lin H, Sveinbjornsson G, Fatemifar G*, et al.* Genome-wide association and Mendelian randomisation analysis provide insights into the pathogenesis of heart failure. *Nat Commun* 2020;**11**:163. doi: 10.1038/s41467-019-13690-5

2. Nielsen JB, Thorolfsdottir RB, Fritsche LG, Zhou W, Skov MW, Graham SE*, et al.* Biobank-driven genomic discovery yields new insight into atrial fibrillation biology. *Nat Genet* 2018;**50**:1234-1239. doi: 10.1038/s41588-018-0171-3

3. Pulit SL, Stoneman C, Morris AP, Wood AR, Glastonbury CA, Tyrrell J*, et al.* Meta-analysis of genome-wide association studies for body fat distribution in 694 649 individuals of European ancestry. *Hum Mol Genet* 2019;**28**:166-174. doi: 10.1093/hmg/ddy327

4. Keaton JM, Kamali Z, Xie T, Vaez A, Williams A, Goleva SB*, et al.* Genome-wide analysis in over 1 million individuals of European ancestry yields improved polygenic risk scores for blood pressure traits. *Nat Genet* 2024;**56**:778-791. doi: 10.1038/s41588-024-01714-w

**Supplemental Table 3.** Sensitivity analysis

| **Characteristic** | **Complete case analysis** | **Missing imputation** |
| --- | --- | --- |
|  | **Adjusted HR (95%CI)** | **Adjusted HR (95%CI)** |
| Females | 0.79 (0.71, 0.88) | 0.81 (0.76, 0.85) |
| Age | 1.04 (1.04, 1.04) | 1.04 (1.03, 1.04) |
| White ethnicity | 1.30 (1.06, 1.59) | 1.15 (1.01, 1.31) |
| Most deprived quintile | 1.20 (1.01, 1.42) | 1.04 (0.96, 1.13) |
| BMI | 1.01 (1.00, 1.02) | 1.01 (1.00, 1.01) |
| Smoking  Never smoker  Former smoker  Current smoker | Ref  0.87 (0.70, 1.07)  0.83 (0.67, 1.03) | Ref  1.01 (0.92, 1.10)  1.01 (0.92, 1.11) |
| Alcohol | 1.03 (0.94, 1.13) | 1.02 (0.96, 1.07) |
| Physical activity  Inactive  Gentle  Moderate  Vigorous | Ref  0.84 (0.72, 0.97)  0.85 (0.72, 1.02)  0.86 (0.59, 1.26) | Ref  0.93 (0.88, 0.99)  0.91 (0.83, 0.99)  0.88 (0.67, 1.15) |
| Hypertension | 1.15 (1.03, 1.29) | 1.15 (1.09, 1.22) |
| CKD | 1.15 (1.06, 1.24) | 1.17 (1.11, 1.23) |
| Diabetes | 1.03 (0.94, 1.12) | 1.06 (1.00, 1.12) |
| Sleep apnoea | 1.17 (0.88, 1.56) | 1.15 (0.95, 1.40) |
| COPD | 1.10 (1.01, 1.19) | 1.11 (1.06, 1.17) |
| Ischemic heart disease | 0.99 (0.89, 1.10) | 0.96 (0.90, 1.01) |
| Valvular heart disease | 1.17 (1.06, 1.29) | 1.21 (1.14, 1.28) |
| Dyslipidaemia | 1.06 (0.95, 1.18) | 0.99 (0.93, 1.06) |
| Stroke | 0.92 (0.83, 1.03) | 0.93 (0.87, 0.99) |

HR = hazard ratio; 95%CI = 95% confident interval; BMI = body mass index; CKD = chronic kidney disease; COPD = chronic obstructive pulmonary disease

**Supplemental Table 4.** Results of linkage disequilibrium score regression

| **Variable** | **r_g_** | **p-value** |
| --- | --- | --- |
| BMI | 0.19 | 6.18 ×10^-20^ |
| Systolic BP | 0.14 | 3.90×10^-8^ |
| Diastolic BP | 0.13 | 3.90×10^-8^ |
| Heart failure | 0.57 | 2.30×10^-59^ |
| Time of light activity | -0.11 | 0.41 |
| Time of moderate activity | 0.14 | 0.36 |
| Time of vigorous activity | 0.04 | 0.52 |
| Days moderate activity | -0.01 | 0.65 |
| Days of vigorous activity | 0.01 | 0.72 |
| Ever smoked | 0.07 | 5.31×10^-4^ |
| Current tobacco smoking | 0.08 | 4.54×10^-3^ |
| COPD | 0.14 | 1.00 ×10^-6^ |

BMI = Body Mass Index; BP = Blood Pressure; COPD = chronic obstructive pulmonary disease

**
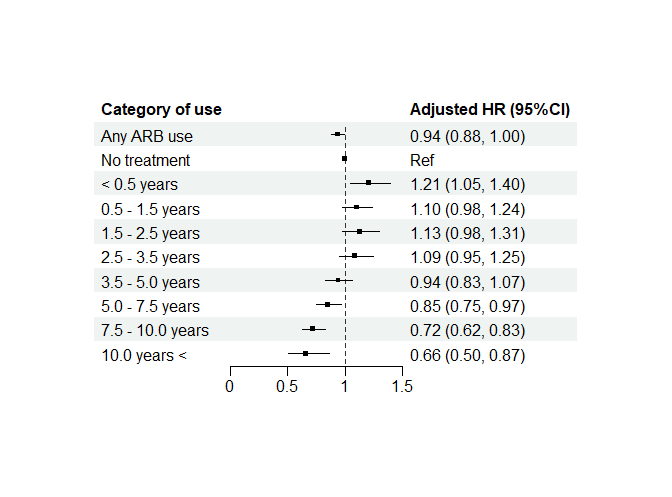

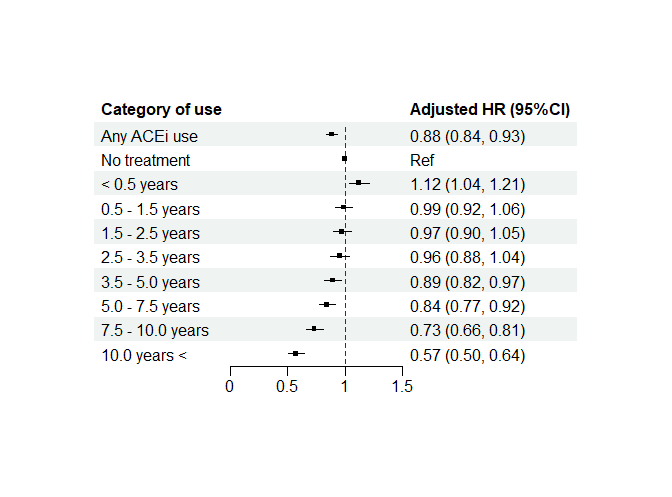
A. B.**

**
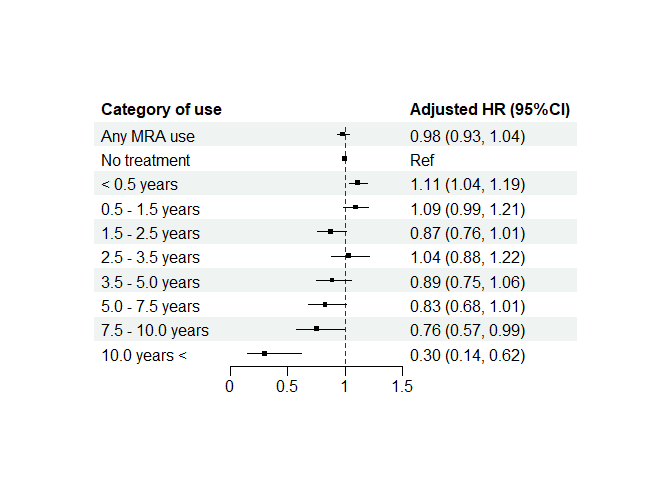
C.**

**Supplemental Figure 1.** Sensitivity analysis of treatment duration on the risk of new-onset AF: A. ACEi; B. ARB; C. MRA.

ACEi = angiotensin-converting enzyme inhibitors; ARB = angiotensin-receptor blockers; MRA = mineralocorticoid receptor antagonists; HR = hazard ratio
